# Supplementary material for: Structures of Marburgvirus glycoprotein and its complex with NPC1 receptor
Source: Nature. 2026 Mar 11;653(8114):621–6. doi: 10.1038/s41586-026-10240-0 (PMC13171430; doi:10.1038/s41586-026-10240-0)
Supplement: Supplementary file 1 — Supplementary Figures 1–3 [file 41586_2026_10240_MOESM1_ESM.pdf]

nature portfolio

https://doi.org/10.1038/s41586-026-10240-0

Supplementary information

Structures of Marburgvirus glycoprotein and its complex with NPC1 receptor

In the format provided by the authors and unedited

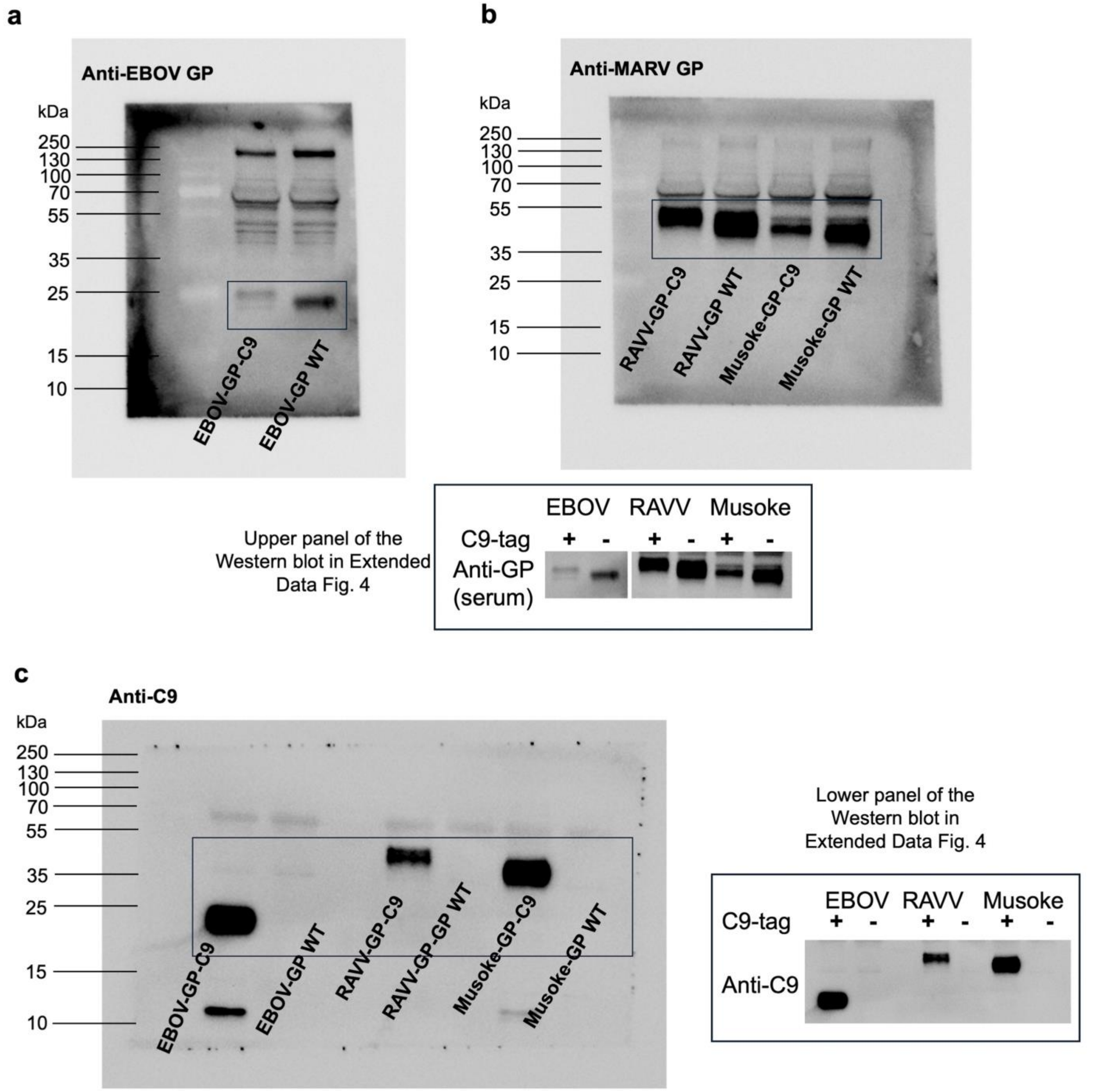

Supplementary Information Figure 1. Uncropped Western blot images corresponding to Extended Data Fig. 4. Boxes mark the regions presented in Extended Data Fig. 4 and are shown alongside the raw images. The boxed bands represent the GP2 fragments of EBOV and MARV. **(a)** All samples were run on the same gel. After SDS-PAGE, proteins were transferred to a membrane and sequentially probed with alpaca anti-EBOV GP serum (1:100) followed by goat anti-alpaca-HRP (1:1000) to detect the GP of EBOV pseudoviruses. **(b)** As in **(a)**, except alpaca anti-MARV GP serum was used as the primary antibody to detect the GP of MARV pseudoviruses. **(c)** As in **(a)**, except a primary mouse anti-C9 antibody and a secondary goat anti-mouse-HRP antibody were used to detect the GP of the indicated pseudovirus.

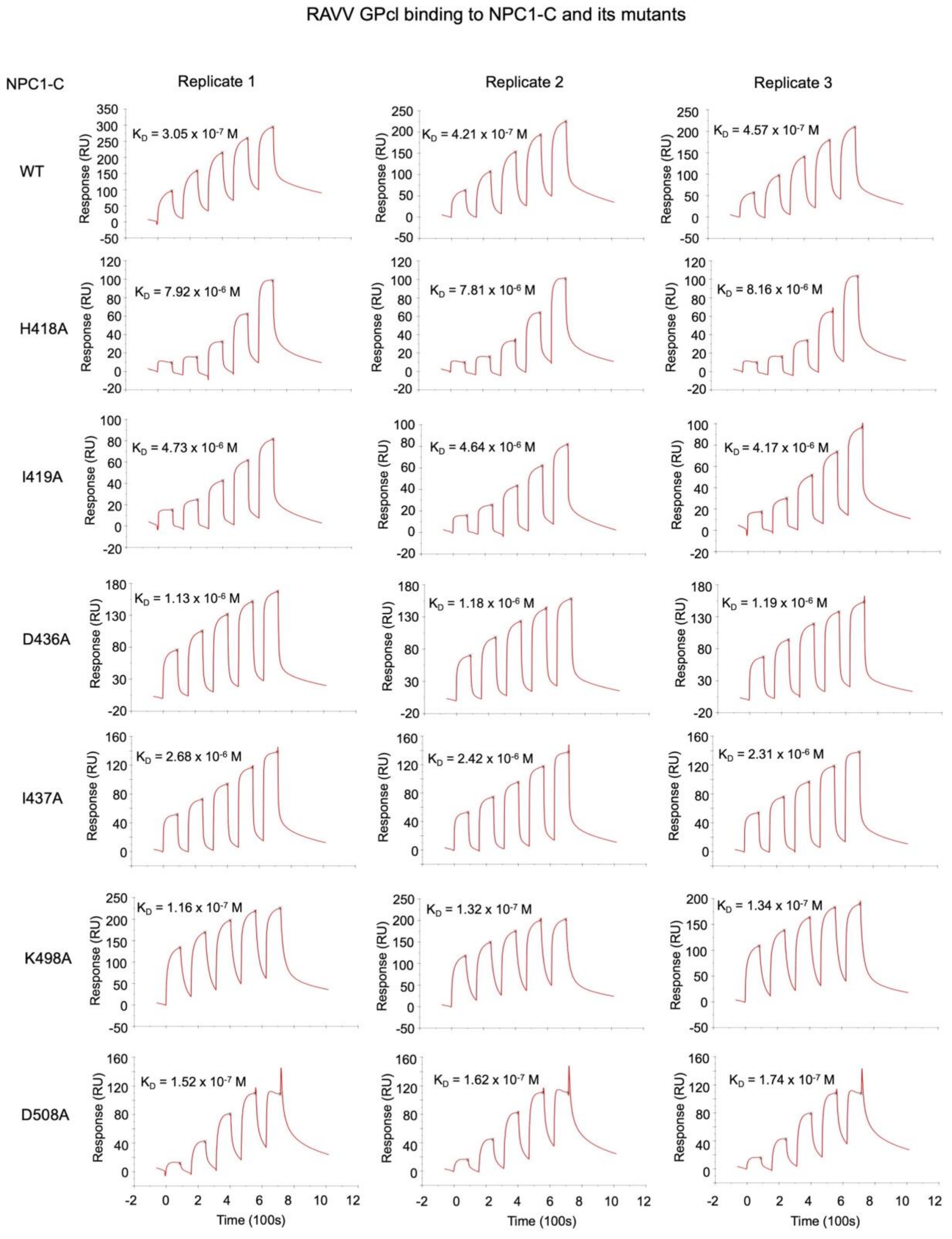

Supplementary Information Figure 2. Full surface plasmon resonance (SPR) curves for binding measurements between RAVV GPcl and NPC1-C or its mutants. For each measurement, curves from three independent replicates are shown. Dissociation constants (K_D) were calculated from these data and are displayed here, but were converted to binding affinities (K_A) in Extended Data Fig. 7d to more clearly illustrate GPcl/NPC1-C binding strengths.

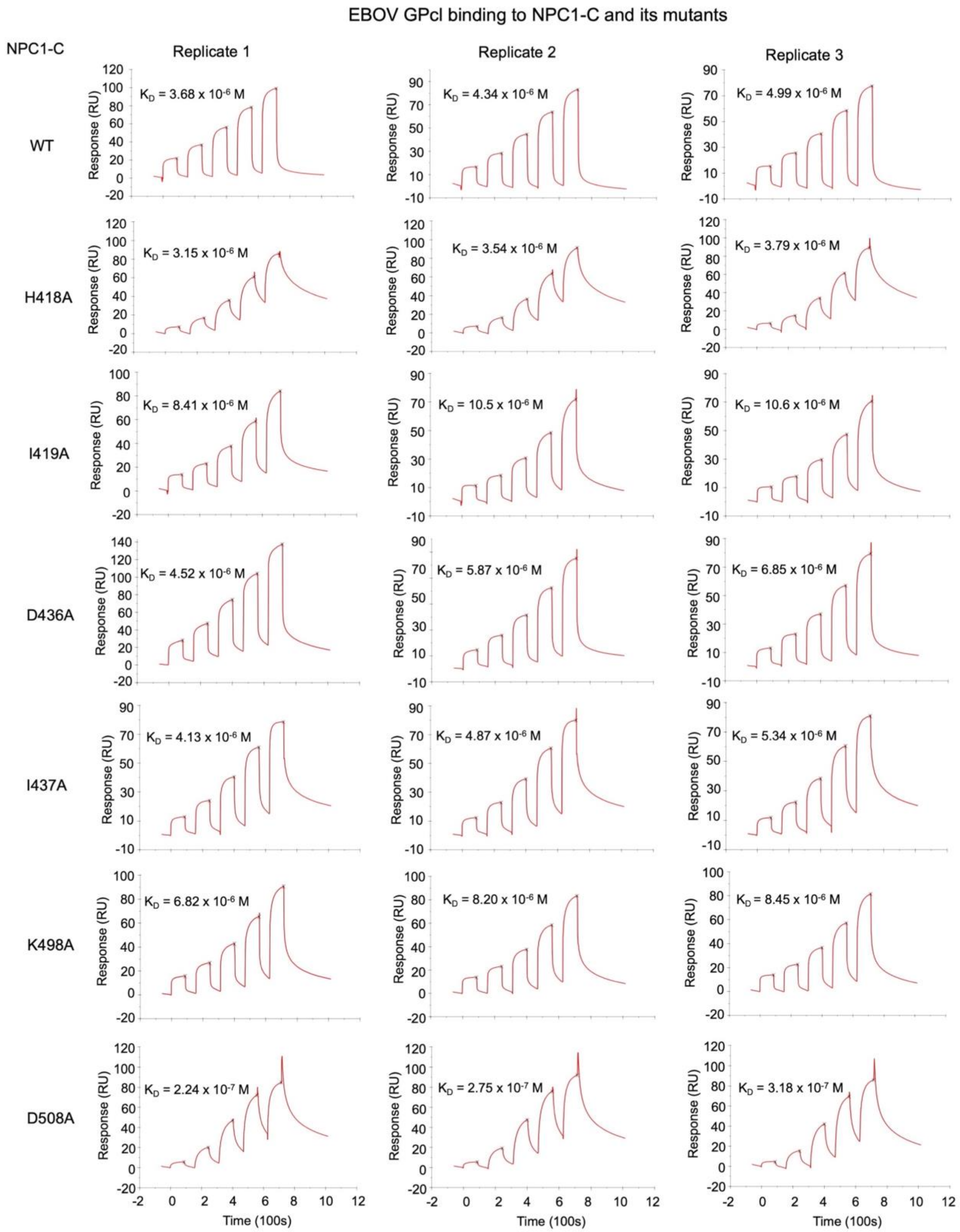

Supplementary Information Figure 3. Full surface plasmon resonance (SPR) curves for binding measurements between EBOV GPcl and NPC1-C or its mutants. For each measurement, curves from three independent replicates are shown. Dissociation constants (K_D) were calculated from these data and are displayed here, but were converted to binding affinities (K_A) in Extended Data Fig. 7d to more clearly illustrate GPcl/NPC1-C binding strengths.
