# Supplementary material for: Structures of Marburgvirus glycoprotein and its complex with NPC1 receptor
Source: Nature. 2026 Mar 11;653(8114):621–6. doi: 10.1038/s41586-026-10240-0 (PMC13171430; doi:10.1038/s41586-026-10240-0)
Supplement: Supplementary file 2 — Reporting Summary [file 41586_2026_10240_MOESM2_ESM.pdf]

nature portfolio

Corresponding author(s): Fang Li, Bin Liu, Gang Ye

Last updated by author(s): Dec 21, 2025

Reporting Summary

Nature Portfolio wishes to improve the reproducibility of the work that we publish. This form provides structure for consistency and transparency in reporting. For further information on Nature Portfolio policies, see our Editorial Policies and the Editorial Policy Checklist.

Statistics

For all statistical analyses, confirm that the following items are present in the figure legend, table legend, main text, or Methods section.

n/a	Confirmed	
☐	☒	The exact sample size (*n*) for each experimental group/condition, given as a discrete number and unit of measurement
☐	☒	A statement on whether measurements were taken from distinct samples or whether the same sample was measured repeatedly
☐	☒	The statistical test(s) used AND whether they are one- or two-sided<br>*Only common tests should be described solely by name; describe more complex techniques in the Methods section.*
☒	☐	A description of all covariates tested
☒	☐	A description of any assumptions or corrections, such as tests of normality and adjustment for multiple comparisons
☐	☒	A full description of the statistical parameters including central tendency (e.g. means) or other basic estimates (e.g. regression coefficient) AND variation (e.g. standard deviation) or associated estimates of uncertainty (e.g. confidence intervals)
☐	☒	For null hypothesis testing, the test statistic (e.g. *F*, *t*, *r*) with confidence intervals, effect sizes, degrees of freedom and *P* value noted<br>*Give P values as exact values whenever suitable.*
☒	☐	For Bayesian analysis, information on the choice of priors and Markov chain Monte Carlo settings
☒	☐	For hierarchical and complex designs, identification of the appropriate level for tests and full reporting of outcomes
☒	☐	Estimates of effect sizes (e.g. Cohen's *d*, Pearson's *r*), indicating how they were calculated

Our web collection on statistics for biologists contains articles on many of the points above.

Software and code

Policy information about availability of computer code

Data collection	EPU version 2.5.
Data analysis	Biacore Evaluation Software v1.1.3; cryoSPARC v4.5.1; MotionCor2; CTFFIND-4.1.13; Coot-0.8.9; Phenix-1.16; UCSF Chimera X v0.93; PyMOL, Version 3.0; LigPlot v.2.2.8; GraphPad Prism v10.4.1; Swiss-Model.

For manuscripts utilizing custom algorithms or software that are central to the research but not yet described in published literature, software must be made available to editors and reviewers. We strongly encourage code deposition in a community repository (e.g. GitHub). See the Nature Portfolio guidelines for submitting code & software for further information.

Data

Policy information about availability of data

All manuscripts must include a data availability statement. This statement should provide the following information, where applicable:

- Accession codes, unique identifiers, or web links for publicly available datasets
- A description of any restrictions on data availability
- For clinical datasets or third party data, please ensure that the statement adheres to our policy

The atomic models and corresponding cryo-EM density maps have been deposited into the PDB and the Electron Microscopy Data Bank, respectively, with accession numbers 9NPS and EMD-49631 (RAVV GPcl), 9NPR and EMD-49630 (RAVV GPcl complexed with NPC1-C), 9NPT and EMD-49632 (RAVV GP-ΔM complexed with Nanosota-MB1).

Research involving human participants, their data, or biological material

Policy information about studies with human participants or human data. See also policy information about sex, gender (identity/presentation), and sexual orientation and race, ethnicity and racism.

Reporting on sex and gender	N/A
Reporting on race, ethnicity, or other socially relevant groupings	N/A
Population characteristics	N/A
Recruitment	N/A
Ethics oversight	N/A

Note that full information on the approval of the study protocol must also be provided in the manuscript.

Field-specific reporting

Please select the one below that is the best fit for your research. If you are not sure, read the appropriate sections before making your selection.

☒ Life sciences ☐ Behavioural & social sciences ☐ Ecological, evolutionary & environmental sciences

For a reference copy of the document with all sections, see nature.com/documents/nr-reporting-summary-flat.pdf

Life sciences study design

All studies must disclose on these points even when the disclosure is negative.

Sample size	No formal statistical methods were used to predetermine sample size. Sample sizes were informed by prior experience with SPR binding experiments and pseudovirus entry assays and were chosen to provide independent replication sufficient to assess the reproducibility of the observed effects.
Data exclusions	No data points were excluded from the analyses.
Replication	The experiments that were repeated, along with the number of replicates, are detailed in the corresponding figure legends.
Randomization	Randomization was not performed because this was an observational study.
Blinding	Investigators were not blinded to group allocation, as this was an observational study.

Reporting for specific materials, systems and methods

We require information from authors about some types of materials, experimental systems and methods used in many studies. Here, indicate whether each material, system or method listed is relevant to your study. If you are not sure if a list item applies to your research, read the appropriate section before selecting a response.

Materials & experimental systems

n/a	Involved in the study	
☐	☒	Antibodies
☐	☒	Eukaryotic cell lines
☒	☐	Palaeontology and archaeology
☒	☐	Animals and other organisms
☒	☐	Clinical data
☒	☐	Dual use research of concern
☒	☐	Plants

Methods

n/a	Involved in the study	
☒	☐	ChIP-seq
☒	☐	Flow cytometry
☒	☐	MRI-based neuroimaging

Antibodies

Antibodies used	Rhodopsin antibody (clone 1D4, sc-57432, Santa Cruz Biotechnology); EBOV GP- and MARV GP–immunized alpaca serum; HRP-conjugated goat anti-alpaca antibody (Jackson ImmunoResearch); HRP-conjugated anti-HA antibody (clone 3F10, 12013819001,

	Sigma-Aldrich).
Validation	Antibodies were purchased from Santa Cruz Biotechnology and were validated by the manufacturer as documented on their website. Additional information for the rhodopsin antibody (clone 1D4, catalog no. sc-57432) is available in the manufacturer's online datasheet.

Eukaryotic cell lines

Policy information about cell lines and Sex and Gender in Research

Cell line source(s)	HEK293T (ATCC, American Type Culture Collection), Huh7 (ATCC), THP-1 (ATCC), HUVEC (ATCC), and Expi293F (Thermo Fisher Scientific) cells.
Authentication	HEK293T, Huh7, and THP-1 cells were authenticated by the vendors using STR profiling. Authentication documentation for Expi293F and HUVEC cells was not available on the vendors' websites.
Mycoplasma contamination	HEK293T cells were tested for mycoplasma contamination in our laboratory and by the vendor and were negative in both cases. Huh7, HUVEC, THP-1, and Expi293F cells were tested and confirmed mycoplasma-negative by the vendors.
Commonly misidentified lines (See ICLAC register)	None reported.

Plants

Seed stocks	N/A
Novel plant genotypes	N/A
Authentication	N/A
